# Genome-Wide Identification of Host Genes Required for Toxicity of Bacterial Cytolethal Distending Toxin in a Yeast Model

**DOI:** 10.3389/fmicb.2019.00890

**Published:** 2019-04-26

**Authors:** Siriyod Denmongkholchai, Prashant Katare, Sarocha Choochuay, Panida Thanyasrisung, Keiko Tsuruda, Motoyuki Sugai, Skorn Mongkolsuk, Oranart Matangkasombut

**Affiliations:** ^1^Interdepartmental Program in Medical Microbiology, Graduate School, Chulalongkorn University, Bangkok, Thailand; ^2^Department of Microbiology and Research Unit on Oral Microbiology and Immunology, Faculty of Dentistry, Chulalongkorn University, Bangkok, Thailand; ^3^Graduate Program in Oral Biology, Faculty of Dentistry, Chulalongkorn University, Bangkok, Thailand; ^4^Laboratory of Biotechnology, Chulabhorn Research Institute, Bangkok, Thailand; ^5^Department of Oral Epidemiology, Graduate School of Biomedical and Health Sciences, Hiroshima University, Hiroshima, Japan; ^6^Department of Antimicrobial Resistance, Graduate School of Biomedical and Health Sciences, Project Research Center for Nosocomial Infectious Diseases (RCNID), Hiroshima University, Hiroshima, Japan; ^7^Antimicrobial Resistance Research Center, National Institute of Infectious Diseases (NIID), Tokyo, Japan

**Keywords:** *Aggregatibacter actinomycetemcomitans*, bacterial genotoxin, cytolethal distending toxin, cytotoxicity, host factors, *Saccharomyces cerevisiae*, yeast model

## Abstract

**Background:**

*Aggregatibacter actinomycetemcomitans*, a periodontal pathogen, secretes a cytolethal distending toxin (AaCDT) that causes host cell cycle arrest and cell death. Although CDT could be an important virulence factor, it is unclear how it enters the nucleus to exert its cytotoxicity.

**Objective:**

To investigate the mechanisms of AaCDT by genome-wide screening for host mutations that confer resistance to the catalytic subunit, AaCdtB, in a *Saccharomyces cerevisiae* model.

**Methods:**

We transformed the yeast haploid deletion library, a collection of yeast mutants with single gene deletions of virtually all non-essential ORFs in the genome, with plasmids carrying galactose-inducible AaCdtB. Yeast mutants that showed resistance to AaCdtB were selected and rescreened by a spotting assay. AaCdtB expression was confirmed by western blot analysis; any mutants that showed no or weak expression of AaCdtB were omitted from the analysis. The lists of genes whose mutations confer resistance to AaCdtB were analyzed for Gene Ontology (GO) term enrichments. Localization of AaCdtB-EGFP was examined using fluorescent microscopy. Nuclear localization relative to EGFP control was calculated and compared to wild-type.

**Results:**

Out of approximately 5,000 deletion mutants, we isolated 243 mutants that are resistant to AaCdtB. GO analyses indicated that genes associated with organic anion transport are significantly enriched (16 genes). Furthermore, several genes associated with the nucleus and endoplasmic reticulum (ER) were identified. Localization studies of AaCdtB, in mutants with the deletion of genes associated with the GO term organic anion transport, showed lower nuclear localization than wild-type. The results suggest that these genes may be required for AaCdtB translocation into the nucleus and its cytotoxicity.

**Conclusion:**

The genome-wide screen in the yeast deletion library allowed us to identify a large number of host genes required for AaCdtB cytotoxicity. Further investigation could lead to more insights into the mechanisms of CdtB intoxication.

## Introduction

Bacterial pathogens often possess several virulence factors to facilitate colonization and survival in hosts. Cytolethal distending toxin (CDT) is a genotoxin produced by many Gram-negative pathogens, such as *Escherichia coli, Aggregatibacter actinomycetemcomitans, Haemophilus ducreyi, Shigella dysenteriae, Helicobacter hepaticus*, and *Campylobacter species* (Gargi et al., 2012; Taieb et al., 2016). CDT induces DNA damages that lead to cell cycle arrest, cellular distension, and cell death in several cell types (Ohara et al., 2004b; Smith and Bayles, 2006; Fais et al., 2016). In particular, lymphocytes undergo rapid apoptosis upon exposure to CDT (Ohara et al., 2004a). Thus, CDT may contribute to pathogenesis by serving as a mechanism for immune evasion, tissue damage, inflammation, and in some cases, carcinogenesis (Fais et al., 2016).

*Aggregatibacter actinomycetemcomitans* (*Aa*) is a periodontal pathogen frequently associated with aggressive periodontitis and produces many virulence factors, including CDT (Herbert et al., 2016). It has been shown that AaCDT could damage gingival and periodontal cells and can induce inflammatory responses (Belibasakis et al., 2004, 2005a,b; DiRienzo, 2014a). Clinical isolates of *Aa* have a high prevalence of CDT (Yamano et al., 2003) and application of CDT alone could lead to gingival inflammation in a rat model (Ohara et al., 2011). These suggest that CDT may be important in *Aa* pathogenicity.

Cytolethal distending toxin is a heterotrimeric AB_2_-type toxin with CdtB as the active catalytic subunit, and CdtA and CdtC as the binding subunits (Lara-Tejero and Galan, 2001). All three subunits of CDT are required for full activity of the toxin. CdtB has homology with DNase-I family, especially in the catalytic residues, and has been shown to cause DNA breaks in target cells and *in vitro*. AaCdtB has also been shown to possess phosphatidylinositol 3,4,5-triphosphate (PIP3) phosphatase activity (Shenker et al., 2007). Nevertheless, we have previously shown that AaCdtB is toxic when expressed in the budding yeast, *Saccharomyces cerevisiae*, which lacks PIP3 (Matangkasombut et al., 2010). This suggests that the DNase activity alone is also sufficient for CdtB toxicity.

AaCDT holotoxin complex is assembled and processed in the periplasm for secretion (Tsuruda et al., 2018). AaCDT is also found in the outer membrane vesicles (OMVs), which could deliver several bacterial proteins into host cells (Thay et al., 2014). The cellular receptors/binding sites of CDT from different pathogens appear to be distinct (Eshraghi et al., 2010). AaCDT interacts with cholesterol in membrane lipid rafts (Boesze-Battaglia et al., 2009) and glycosphingolipid (Mise et al., 2005). Subsequently, CdtB requires retrograde translocation through the golgi and endoplasmic reticulum (ER) into the nucleus, in order to damage host DNA (DiRienzo, 2014b; Guerra et al., 2005). However, there is a significant gap in our knowledge regarding how CdtB is transported from the ER to the nucleus. A non-canonical nuclear localization signal (NLS) has been identified in the N-terminus of AaCdtB and is shown to be required for its toxicity (Nishikubo et al., 2003). The NLS is also necessary for AaCdtB toxicity in the yeast model (Matangkasombut et al., 2010). This suggests that AaCdtB uses a similar mode of translocation into the nucleus of yeast cells, and that yeast could be a good platform to further dissect the mechanisms of AaCdtB translocation and cytotoxicity.

In addition, it has been observed that *in vitro* DNase activity of CdtB was rather low in comparison to its ability to induce DNA damage *in vivo* (Elwell and Dreyfus, 2000; Lara-Tejero and Galan, 2000; Gargi et al., 2012). This discrepancy suggests that host factors may be required to facilitate CdtB cytotoxicity. However, limited information exists in this regard. Therefore, through taking advantage of the yeast deletion library (Giaever and Nislow, 2014), we aimed to systematically identify host factors that may play a role in CdtB translocation and cytotoxicity by performing a genome-wide screen for mutations that confer AaCdtB resistance.

## Materials and Methods

### Yeast Culture and Growth Conditions

The *MATa* haploid yeast deletion library with BY4741 background (*MATa his3*Δ*1 leu2*Δ*0 met15*Δ*0 ura3*Δ*0*) was purchased from Invitrogen, United States. The library was transformed with pYES2-CdtB, a plasmid carrying AaCdtB under galactose-inducible promoter [described in Matangkasombut et al. (2010)], in 96-well plates following a protocol previously described (Voynov et al., 2006). Briefly, yeast cultures were incubated with PEG/LiOAc/TE/ssDNA mixture for 3 h at 30°C, and heat shocked for 45 min at 42°C. Transformants were selected first in liquid synthetic complete media (SC) lacking uracil (SC-Ura) (ingredients from Sigma-Aldrich, United States) for 3 days and on solid SC-Ura for 2 days. Any mutants that failed to transform in batch were individually transformed using standard protocol (Gietz and Woods, 2002).

### CdtB Susceptibility Screening

The transformed yeast library was grown overnight in SC-Ura supplemented with 2% glucose. The cultures were serially diluted and inoculated with a 96-pin floating pin replicator (V&P Scientific, Inc., United States) on SC-Ura media containing 2% galactose (inducing media) and on that containing 2% glucose (repressing media) as a control. Mutants showing significant growth on galactose plates, in the dilutions where the wild-type yeast strain BY4741 did not grow, were selected for further confirmatory screening.

Subsequently, the mutants selected in the primary screen were compiled in 96-well plates and rescreened by spotting dilutions of cultures onto glucose and galactose plates. Mutants that grew well on galactose plates were further analyzed by spot tests as described (Matangkasombut et al., 2010). Briefly, log phase cultures were serially diluted and spotted on inducing and repressing media. After 40–48 h of incubation at 30°C, yeast growth was observed. Mutants that showed better growth than wild-type on the galactose plate were selected for rescreening. After at least four repeats, mutants that consistently show resistance to CdtB were classified as CdtB resistant mutants.

### Immunoblot Assay for AaCdtB Expression

CdtB expression in CdtB resistant mutants was examined using an immunoblot assay. CdtB resistant mutants were grown to log phase and galactose was added to 2% to induce CdtB expression. Whole cell lysates were extracted using glass beads and trichloroacetic acid (Keogh et al., 2006). The samples were resolved using SDS-PAGE and transferred onto nitrocellulose membranes. Immunoblot was performed using Rabbit anti-AaCdtB serum (Sugai et al., 1998) and goat anti-rabbit IgG antibody, conjugated with HRP to detect CdtB protein in the whole cell extract.

### Gene Ontology (GO) Analyses

The list of genes whose deletion conferred CdtB resistance from our genome-wide screen was compiled and analyzed using web-based tools available on the *Saccharomyces* genome database (SGD). GO Slim Mapper^1^ was used to map the gene list to broad GO terms (Yeast-GO slim) related to biological processes, molecular functions, and cellular components. In addition, GO Term Finder^2^ was used to search for GO terms that are shared among the genes in the list where *p* < 0.05 is considered significant.

### Nuclear Localization of CdtB-EGFP Fusion Protein

The plasmid carrying CdtB-EGFP fusion was constructed by inserting a Klenow-treated *NotI-XbaI cdtB*-containing fragment from pYES-CdtB into BamHI-XbaI sites of a pYES2-EGFP plasmid (a gift from Prof. C. Boonchird, Mahidol University, Thailand) by blunt-end ligation. A 11-amino acid linker was inserted between the CdtB and EGFP open reading frames. After transformation into BY4741, CdtB-EGFP protein expression was confirmed by immunoblotting and using the CdtB susceptibility spot assay (Supplementary Figure 1).

For the protein localization study, yeast cultures were transformed with pYES2-CdtB-EGFP and grown to mid-log phase. After CdtB induction by growing in media containing 2% galactose for 8 h, cells were fixed in 3.7% formaldehyde in PBS buffer (pH7.4) for 15 min and permeabilized with 1% triton X-100 at 42°C for 30 min. Samples were incubated with 0.1 μg/ml DAPI for 5 min for yeast nuclei staining. Cells were washed twice with PBS and dropped on 1% agarose patch on glass slides. Fluorescent images were captured with confocal fluorescent microscope (Olympus, FV10i-ASW model) at 60× optical magnification.

Fluorescent images were analyzed by Mander’s coefficient function of Just Another Co-localization Plug-in (JACoP) in the ImageJ program (Bolte and Cordelieres, 2006). The mean fluorescent intensity and standard deviation (SD) for each cell were calculated for DAPI and EGFP channels. The threshold level for the positive signal of DAPI was set at ≥ mean+1SD, while that of EGFP was set at ≥ mean+2SD. For each cell, we first defined the DAPI-positive area as the nucleus, and counted the pixel number with EGFP signal above the threshold. Then, we calculated the relative percentage of nuclear localization of CdtB by comparing the percentage of EGFP-positive pixel numbers in nucleus over the total in yeast cells expressing CdtB-EGFP vs. EGFP control. The data was analyzed using Mann-Whitney U test with *p* < 0.05 considered statistically significant.

## Results

### Genome-Wide Screen Identified Gene Deletions That Confer CdtB Resistance

**FIGURE 1:**
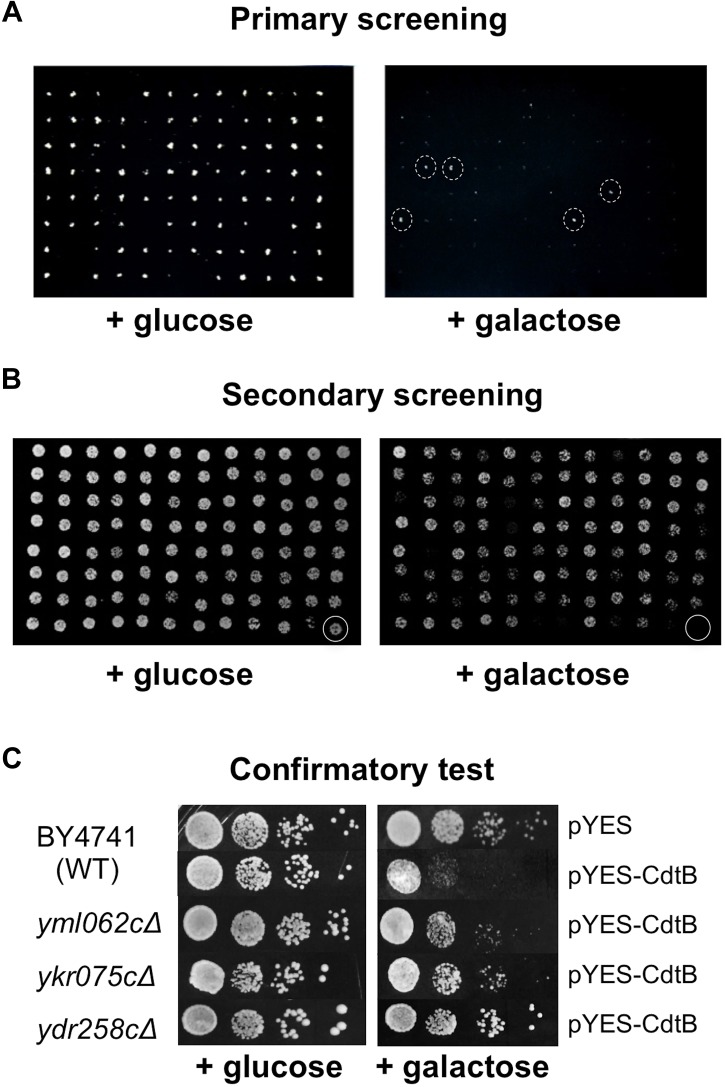
Screening of the yeast deletion library for mutations that confer CdtB resistance. Representative images of yeast deletion mutants carrying pYES-CdtB spotted on glucose (repressing media) and on galactose (inducing media). **(A)** Primary screening of the yeast deletion library was carried out using a 96-pin replicator. An example of the screen of a library plate is shown (library plate no. 4). The mutants with dashed circles were selected for secondary screening. **(B)** Secondary screening was performed by spotting dilutions of mutants that showed resistance in the primary screen. The 513 mutants from the primary screening were compiled in 96-well plates and dilutions of cultures were spotted on solid media. An example of a compilation plate at 1:200 dilution is shown (plate R2). The last well (circled) was the wild-type as a control. **(C)** Confirmatory test was performed using spot tests of 10-fold serial dilutions of mutants in comparison to the wild-type control. Three examples of CdtB resistant mutants that showed better growth than wild-type at various levels are shown. Plates were photographed after approximately 40 h of incubation at 30°C.

Approximately 5,000 non-essential open reading frames (ORFs) from the yeast deletion library were successfully transformed with *p*YES-CdtB and were screened for CdtB susceptibility. From the primary screening, we identified 513 deletion mutants that showed growth on 2% galactose (CdtB inducing media) in the dilutions where most mutants did not grow (Figure 1A). These were compiled in 96-well plates and dilutions were spotted on solid media with 2% galactose and with 2% glucose (repressing media) (Figure 1B). Mutants that showed growth on a galactose plate, at the dilution where the wild-type did not grow, were selected for further confirmation. The CdtB resistant phenotype was confirmed by spotting serial dilutions of cultures for CdtB susceptibility tests (Figure 1C). When comparing the growth on galactose with that on glucose, CdtB expression leads to reduction of both the number of colonies and colony size in wild-type yeast (Figure 1C). We previously showed, using survival plating assays to quantify the effect of CdtB, that CdtB expression reduces the number of surviving colonies by approximately 50% in wild-type yeast (Matangkasombut et al., 2010). Different degrees of CdtB expression led to dose-dependent effects on yeast growth. These could be observed by both survival plating and plate sensitivity (spot) assay (Supplementary Figures 2A–C). However, the plate sensitivity assay allows us to better observe the overall effect of CdtB, including the effect on colony size. Since CdtB-induced cell cycle arrest can lead to a small colony size, formation of larger colonies on galactose media also reflects resistance to the effect of CdtB. The effect of CdtB could be more clearly observed at an early time after incubation (approximately 40 h) (Supplementary Figure 2D). In addition, since various gene deletion mutants may grow at different rates, we examined the effect of CdtB by comparing the degrees of growth reduction on media with galactose vs. with glucose of the mutants to that of the wild-type (Figure 1C).

Altogether, we identified 281 mutants that consistently showed better growth than wild-type at various levels, in terms of number of colonies and colony size, in at least four repeats (Supplementary Figure 3). To rule out the possibility of resistance due to defect in CdtB expression, whole cell extracts of these 281 mutants were examined by anti-CdtB immunoblot assay (data not shown). Mutants that showed no or weak expressions of CdtB were omitted from further analysis. Finally, 243 mutants were classified as CdtB resistant (Supplementary Table 1).

### Gene Ontology (GO) Analysis of Genes Required for CdtB Toxicity

**FIGURE 2:**
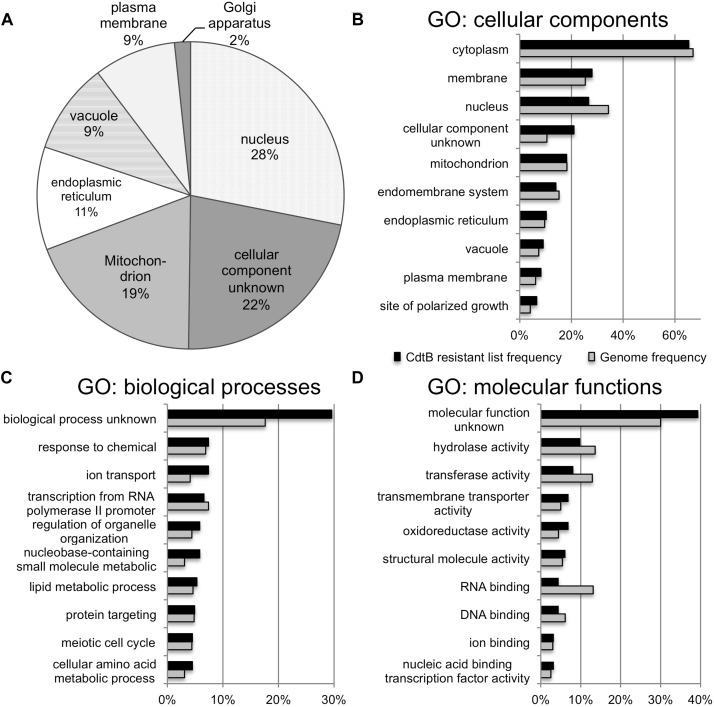
GO analyses of the list of genes whose deletion lead to CdtB resistance. **(A)** Proportion of genes in the list associated with various cellular components. Top 10 GO terms associated with the CdtB resistant list (black bars) in comparison to the genome frequency (gray bars) in the categories of cellular components **(B)**, biological processes **(C)**, and molecular functions **(D)**.

The list of genes whose deletion confers CdtB resistance (243 genes) was analyzed by GO Slim Mapper tool on SGD. These genes have been annotated to localize to a variety of cellular components (Figure 2A). The top 10 broad GO terms identified according to the 3 categories: cellular components, biological processes, and molecular functions, are summarized in Figure 2B–D, respectively.

Further analysis using GO Term Finder identified “organic anion transport (OAT)” (GO:0015711) to be significantly enriched in the gene list, as compared to the genome frequency in the biological process category (*p*-value = 0.03846). There are 16 genes in the list of 243 CdtB resistant genes (6.6%) with this GO term annotation, while the genome frequency was 135 in 7165 genes (1.9%). These 16 genes are listed in Table 1. We searched for GO terms associated with these 16 genes in relation to cellular components and found that most genes are localized in the membrane part (15 genes), vacuole (8 genes) and vacuolar membrane (6 genes). When we searched for GO terms related to molecular functions, most genes show transporter activity (14 genes) and terms related to transmembrane transporter activity (9 genes). These results suggest that these 16 genes, that localize to membrane/vacuole and function as transporters, may play an important role in CdtB cytotoxicity.

**Table 1:** List of 16 organic anion transport genes that showed CdtB resistant phenotype.

Classification	Systematic name	Standard name	Description from SGD
Fatty acid transport	YBR041W	*FAT1*	Very long chain fatty acyl-CoA synthetase and fatty acid transporter; activates imported fatty acids with a preference for very long lengths (C20-C26); has a separate function in the transport of long chain fatty acids
	YOR049C	*RSB1*	Putative sphingoid long-chain base (LCB) efflux transporter; integral membrane transporter that localizes to the plasma membrane and may transport long chain bases (LCBs) from the cytoplasmic side toward the extracytoplasmic side of the membrane; role in glycerophospholipid translocation; suppressor of the sphingoid LCB sensitivity of an LCB-lyase mutation
	YLR193C	*UPS1*	Phosphatidic acid transfer protein; plays a role in phospholipid metabolism by transporting phosphatidic acid from the outer to the inner mitochondrial membrane; localizes to the mitochondrial intermembrane space; null mutant has altered cardiolipin and phosphatidic acid levels; ortholog of human PRELI
	YNL264C	*PDR17*	Phosphatidylinositol transfer protein (PITP); downregulates Plb1p-mediated turnover of phosphatidylcholine; forms a complex with Psd2p which appears essential for maintenance of vacuolar PE levels; found in the cytosol and microsomes; homologous to Pdr16p; deletion affects phospholipid composition
	YJL145W	*SFH5*	Non-classical phosphatidylinositol transfer protein (PITP); exhibits PI- but not PC-transfer activity; localizes to the peripheral endoplasmic reticulum(ER), cytosol and microsomes; similar to Sec14p; partially re-localizes to the plasma membrane upon DNA replication stress
Amino acid and protein transport	YMR088C	*VBA1*	Permease of basic amino acids in the vacuolar membrane
	YCL069W	*VBA3*	Permease of basic amino acids in the vacuolar membrane; VBA3 has a paralog, VBA5, that arose from a segmental duplication
	YGR206W	*MVB12*	ESCRT-I subunit required to stabilize ESCRT-I core complex oligomers; the ESCRT-I core complex (Stp22p, Vps28p, Srn2p) is involved in ubiquitin-dependent sorting of proteins into the endosome; deletion mutant is sensitive to rapamycin and nystatin
	YPR058W	*YMC1*	Secondary mitochondrial inner membrane glycine transporter; required with HEM25 for the transport of glycine into the mitochondria for the initiation of heme biosynthesis; proposed role in oleate metabolism and glutamate biosynthesis; member of the mitochondrial carrier (MCF) family; localizes to the vacuole in response to H2O2; YMC1 has a paralog, YMC2, that arose from the whole genome duplication
	YPR149W	*NCE102*	Protein of unknown function; contains transmembrane domains; involved in secretion of proteins that lack classical secretory signal sequences; component of the detergent-insoluble glycolipid-enriched complexes (DIGs); NCE102 has a paralog, FHN1, that arose from the whole genome duplication
	YOR130C	*ORT1*	Ornithine transporter of the mitochondrial inner membrane; exports ornithine from mitochondria as part of arginine biosynthesis; functionally complemented by human ortholog, SLC25A15, which is associated with hyperornithinaemia-hyperammonaemia-homocitrullinuria (HHH) syndrome, but HHH-associated variants fail to complement
	YER119C	*AVT6*	Vacuolar aspartate and glutamate exporter; member of a family of seven genes (AVT1-7) related to vesicular GABA-glycine transporters; involved in compartmentalizing acidic amino acids in response to nitrogen starvation; AVT6 has a paralog, AVT5, that arose from the whole genome duplication
Carbohydrate transport	YKL217W	*JEN1*	Monocarboxylate/proton symporter of the plasma membrane; transport activity is dependent on the pH gradient across the membrane; mediates high-affinity uptake of carbon sources lactate, pyruvate, and acetate, and of the micronutrient selenite, whose structure mimics that of monocarboxylates; expression and localization are tightly regulated, with transcription repression, mRNA degradation, and protein endocytosis and degradation all occurring in the presence of glucose
Pyruvate transport	YHR162W	*MPC2*	Highly conserved subunit of the mitochondrial pyruvate carrier (MPC); expressed during growth on fermentable carbon sources, and heterodimerizes with Mpc1p to form the fermentative isoform of MPC; MPC localizes to the mitochondrial inner membrane and mediates pyruvate uptake; MPC2 paralog, MPC3, heterodimerizes with Mpc1p to form the respiratory MPC isoform
FAD transport	YGL139W	*FLC3*	Putative FAD transporter, similar to Flc1p and Flc2p; localized to the ER; FLC3 has a paralog, FLC1, that arose from the whole genome duplication
Ion transport	YNR013C	*PHO91*	Low-affinity vacuolar phosphate transporter; exports phosphate from the vacuolar lumen to the cytosol; regulates phosphate and polyphosphate metabolism; acts upstream of Pho81p in regulation of the PHO pathway; localizes to sites of contact between the vacuole and mitochondria (vCLAMPs); deletion of pho84, pho87, pho89, pho90, and pho91 causes synthetic lethality; transcription independent of Pi and Pho4p activity; overexpression results in vigorous growth

Since a significant gap in our knowledge on intracellular translocation of CdtB is with regards to how CdtB moves from the ER to the nucleus, we identified genes in the CdtB resistant list that localize to the ER. From our search, 25 genes showed ER localization (Table 2). Among these, 12 genes localize to the ER membrane. Interestingly, *HRD3*, which encodes an ER membrane protein that plays a central role in ER-associated protein degradation (ERAD), was also included in this list.

**Table 2:** CdtB resistant genes that associate with ER.

Systematic name	Standard name	Description from SGD
*YBL082C*	*ALG3*	Dolichol-P-Man dependent alpha (1–3) mannosyltransferase; involved in synthesis of dolichol-linked oligosaccharide donor for N-linked glycosylation of proteins; G353A missense mutation in human ortholog ALG3 implicated in carbohydrate deficient glycoprotein syndrome type IV, which is characterized by microcephaly, severe epilepsy, minimal psychomotor development and partial deficiency of sialic acids in serum glycoproteins; wild-type human *ALG3* can complement yeast *alg3* mutant
*YBR041W*	*FAT1*	Very long chain fatty acyl-CoA synthetase and fatty acid transporter; activates imported fatty acids with a preference for very long lengths (C20–C26); has a separate function in the transport of long chain fatty acids
*YBR130C*	*SHE3*	Protein adaptor between Myo4p and the She2p-mRNA complex; part of the mRNA localization machinery that restricts accumulation of certain proteins to the bud; also required for cortical ER inheritance
*YCL069W*	*VBA3*	Permease of basic amino acids in the vacuolar membrane; *VBA3* has a paralog, *VBA5*, that arose from a segmental duplication
*YDL122W*	*UBP1*	Ubiquitin-specific protease; removes ubiquitin from ubiquitinated proteins; cleaves at the C terminus of ubiquitin fusions irrespective of their size; capable of cleaving polyubiquitin chains
*YDL204W*	*RTN2*	Reticulon protein; involved in nuclear pore assembly and maintenance of tubular ER morphology; promotes membrane curvature; regulates the ER asymmetry-induced inheritance block during ER stress; role in ER-derived peroxisomal biogenesis; interacts with Sec6p, Yip3p, and Sbh1p; less abundant than *RTN1*; member of *RTNLA* (reticulon-like A) subfamily; protein increases in abundance and re-localizes to plasma membrane upon DNA replication stress
*YDR504C*	*SPG3*	Protein required for high temperature survival during stationary phase; not required for growth on non-fermentable carbon sources; SWAT-GFP and mCherry fusion proteins localize to the ER
*YEL016C*	*NPP2*	Nucleotide pyrophosphatase/phosphodiesterase; mediates extracellular nucleotide phosphate hydrolysis along with Npp1p and Pho5p; activity and expression enhanced during conditions of phosphate starvation; involved in spore wall assembly; SWAT-GFP and mCherry fusion proteins localize to the ER; *NPP2* has a paralog, *NPP1*, that arose from the whole genome duplication; *npp1 npp2* double mutant exhibits reduced dityrosine fluorescence relative to single mutants
*YGL032C*	*AGA2*	Adhesion subunit of a-agglutinin of a-cells; C-terminal sequence acts as a ligand for alpha-agglutinin (Sag1p) during agglutination, modified with O-linked oligomannosyl chains, linked to anchorage subunit Aga1p via two disulfide bonds
*YGL139W*	*FLC3*	Putative FAD transporter, similar to Flc1p and Flc2p; localized to the ER; FLC3 has a paralog, FLC1, that arose from the whole genome duplication
*YGR263C*	*SAY1*	Sterol deacetylase; component of the sterol acetylation/deacetylation cycle along with Atf2p; active both in the ER and in lipid droplets; integral membrane protein with active site in the ER lumen; green fluorescent protein (GFP)-fusion protein localizes to the ER
*YHL044W*		Putative integral membrane protein; member of DUP240 gene family; green fluorescent protein (GFP)-fusion protein localizes to the plasma membrane in a punctate pattern
*YIL023C*	*YKE4*	Zinc transporter; localizes to the ER; null mutant is sensitive to calcofluor white, leads to zinc accumulation in cytosol; ortholog of the mouse KE4 and member of the ZIP (ZRT, IRT-like Protein) family
*YJL134W*	*LCB3*	Long-chain base-1-phosphate phosphatase; specific for dihydrosphingosine-1-phosphate, regulates ceramide and long-chain base phosphate levels, involved in incorporation of exogenous long chain bases in sphingolipids; *LCB3* has a paralog, *YSR3*, that arose from the whole genome duplication
*YJL145W*	*SFH5*	Non-classical phosphatidylinositol transfer protein (PITP); exhibits PI- but not PC-transfer activity; localizes to the peripheral ER, cytosol and microsomes; similar to Sec14p; partially re-localizes to the plasma membrane upon DNA replication stress
*YJR010C-A*	*SPC1*	Subunit of the signal peptidase complex (SPC); SPC cleaves the signal sequence from proteins targeted to the ER; homolog of the *SPC12* subunit of mammalian signal peptidase complex; protein abundance increases in response to DNA replication stress
*YLR207W*	*HRD3*	ER membrane protein that plays a central role in ERAD; forms HRD complex with Hrd1p and ER-associated protein degradation (ERAD) determinants that engages in lumen to cytosol communication and coordination of ERAD events
*YML128C*	*MSC1*	Protein of unknown function; mutant is defective in directing meiotic recombination events to homologous chromatids; the authentic, non-tagged protein is detected in highly purified mitochondria and is phosphorylated
*YNL012W*	*SPO1*	Meiosis-specific prospore protein; required for meiotic spindle pole body duplication and separation; required to produce bending force necessary for proper prospore membrane assembly during sporulation; has similarity to phospholipase B
*YNR075W*	*COS10*	Endosomal protein involved in turnover of plasma membrane proteins; member of the *DUP380* subfamily of conserved, often sub-telomeric *COS* genes; required for the multivesicular vesicle body sorting pathway that internalizes plasma membrane proteins for degradation; Cos proteins provide ubiquitin in trans for non-ubiquitinated cargo proteins
*YOR049C*	*RSB1*	Putative sphingoid long-chain base (LCB) efflux transporter; integral membrane transporter that localizes to the plasma membrane and may transport long chain bases (LCBs) from the cytoplasmic side toward the extra-cytoplasmic side of the membrane; role in glycerophospholipid translocation; suppressor of the sphingoid LCB sensitivity of an LCB-lyase mutation
*YPL154C*	*PEP4*	Vacuolar aspartyl protease (proteinase A); required for post-translational precursor maturation of vacuolar proteinases; important for protein turnover after oxidative damage; plays a protective role in acetic acid induced apoptosis; synthesized as a zymogen, self-activates
*YPR063C*		ER-localized protein of unknown function
*YPR109W*	*GLD1*	Predicted membrane protein; SWAT-GFP and mCherry fusion proteins localize to the ER; diploid deletion strain has high budding index
*YPR149W*	*NCE 102*	Protein of unknown function; contains transmembrane domains; involved in secretion of proteins that lack classical secretory signal sequences; component of the detergent-insoluble glycolipid-enriched complexes (DIGs); *NCE102* has a paralog, *FHN1*, that arose from the whole genome duplication

### CdtB Localization in Organic Anion Transport Mutants

**FIGURE 3:**
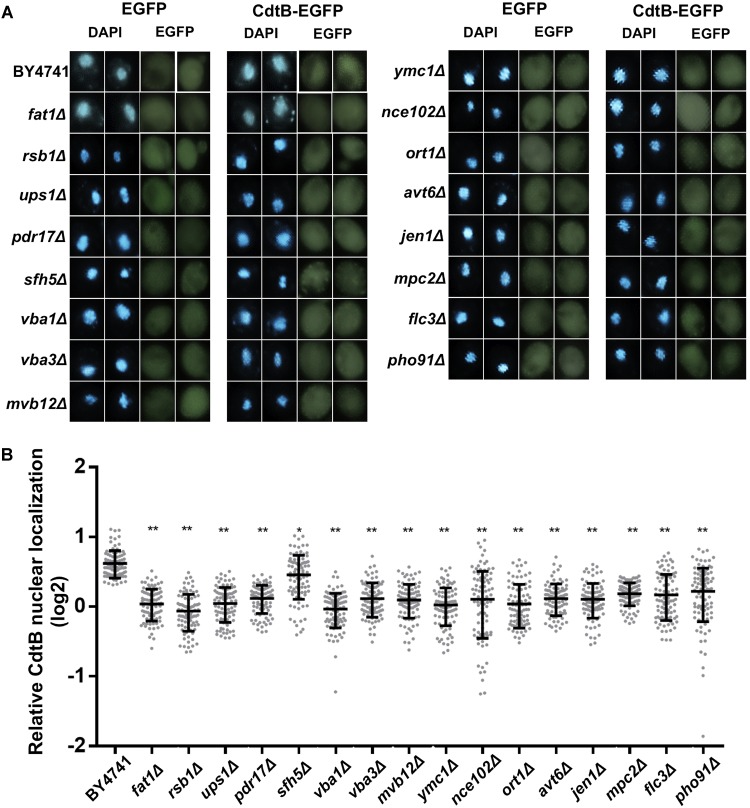
CdtB nuclear localization was reduced in mutants associated with organic anion transport. **(A)** Representative fluorescent images (at 600 magnification) of localization of CdtB-EGFP and EGFP control in wild-type and mutants associated with the GO term organic anion transport are shown. DAPI was used to stain the yeast nuclei. Two examples of each are shown **(B)** Ratio of nuclear localization of CdtB-EGFP relative to EGFP control was calculated using Mander’s coefficient. Thirty cells were randomly selected for each samples in each experiment (total *N* = 90) and Mann-Whitney U test was used to analyze the data. ^∗^*p* < 0.001, ^∗∗^*p* < 0.0001.

As we found an enrichment in genes related to organic anion transport in the CdtB resistant list, we hypothesized that the mutations may disrupt CdtB translocation into the nucleus. We used CdtB-EGFP fusion protein as a tool to examine CdtB localization in these 16 mutants. After inducing CdtB-EGFP expression, fluorescent signals were observed using a confocal fluorescent microscope. The results are shown in Figure 3A. CdtB nuclear localization was evaluated by Mander’s coefficient in comparison to the EGFP vector control. The results showed that CdtB nuclear localization in all mutants are lower than that in the wild-type, as shown in Figure 3B.

## Discussion

In this study, we employed the yeast deletion library to systemically screen for genes whose deletion confers resistance to AaCdtB. We identified 243 genes with diverse roles, localized in different cellular components, that may be required for AaCdtB toxicity. We hypothesized that these genes may be required for CdtB to translocate into the nucleus or to act on its DNA substrates.

After CDT binds to host membrane, previous studies showed that CdtB is internalized and translocated to the nucleus (Guerra et al., 2005; Gargi et al., 2012; DiRienzo, 2014b). The fact that the previously identified NLS was also required for yeast cytotoxicity suggests that AaCdtB uses similar mechanisms to gain access to the yeast nucleus (Matangkasombut et al., 2010). Thus, although AaCdtB was expressed inside the cells in yeast, this model system can still be used to examine the mechanisms of CdtB toxicity in the processes after internalization, which is where an important gap of our current understanding of CdtB mechanisms lies. The availability of the yeast deletion library makes it possible to systemically investigate the requirement of each gene in the genome for CdtB toxicity in an unbiased manner (Giaever and Nislow, 2014). Previous screens of CdtB in the yeast deletion library were performed to identify deletions that confer hypersensitivity to CdtB from *Campylobacter jejuni* (CjCdtB) (Kitagawa et al., 2007; Guerra et al., 2011). These screens identified genes required for cell survival upon CdtB intoxication, such as genes related to DNA repair pathways and cytoskeleton remodeling. Thus, this is the first study that used the yeast model to identify genes that facilitate AaCdtB toxicity.

A previous report using insertional mutagenesis in haploid human cells identified mutations in 12 genes that are differentially required for toxicity of CDTs from 4 different bacterial species (Carette et al., 2011). AaCDT was shown to require sphingomyelin synthase 1 (SGMS1), synaptogyrin 2 (SYNGR2), Golgi glycoprotein 1 (GLG1) and the vacuolar ATPase subunit 2 (ATP6V0A2), which are membrane proteins in the plasma membrane and/or in the endomembrane system (Carette et al., 2011). While this list may include proteins involved in membrane binding and internalization, our study focused on the process that happens after internalization. Indeed, we showed that CdtB localization to the nucleus was disrupted in the mutants associated with organic anion transport process. In addition, several ER-associated genes were identified in our screen; especially interesting is *HRD3*, a component of the ER-associated protein degradation (ERAD) pathway. ERAD pathway has previously been implicated in host cell entry and toxicity of several bacterial toxins, including AaCDT and *Haemophilus ducreyi* CDT (HdCDT), but not CjCDT (Eshraghi et al., 2014). We speculate that the deletions of ERAD component genes would have a similar effect on HdCDT, but this requires further tests. Together, our results added to the current knowledge and further suggest that the functions of the organic anion transport process and endomembrane system play important roles in facilitating CdtB translocation and cytotoxicity.

Furthermore, 65 genes localized to the nucleus were identified in this screen. These genes are of particular interest since it is unknown if CdtB requires interactions with any host proteins or cofactors in order to damage the DNA. Genes with functions related to DNA metabolism and chromatin may be involved in the interaction of CdtB with DNA. In addition, the genes identified in this study have diverse cellular functions. How these different processes in the nucleus could affect CdtB toxicity requires further investigations. Although our screen was performed in yeast, several of the genes identified have orthologs in human. Thus, this study provides additional candidates to be further tested in human cells.

The screening procedures used in this study still carry certain limitations. Since the primary screening was based on growth on galactose media, mutants that do not grow well on galactose or have slow growth phenotype would not be detected in the first screen. Thus, it is possible that CdtB may also require other genes besides those found in this screen. In addition, further investigations on each gene of interest are required to determine if the effect of the gene deletion on CdtB resistance is direct or indirect.

## Conclusion

In conclusion, we identified 243 genes that may play a role in facilitating AaCdtB cytotoxicity. The diverse functions of these genes suggest that CdtB requires complex interactions with host factors in order to translocate into the nucleus and damage host DNA. Further understanding of the mechanisms of CdtB may help in developing novel strategies to hinder its activity or to better exploit its activity in targeting cancer cells (Lai et al., 2016).

## Data Availability

All datasets generated for this study are included in the manuscript and/or the Supplementary Files.

## Author Contributions

OM, MS, PT, KT, and SM contributed conception and design of the study. OM, SD, PK, and SC performed experiments and initial analysis. OM and SD analyzed data and wrote the manuscript. All authors contributed to manuscript revision, read and approved the submitted version.

## Conflict of Interest Statement

The authors declare that the research was conducted in the absence of any commercial or financial relationships that could be construed as a potential conflict of interest.

## Footnotes

1 https://www.yeastgenome.org/cgi-bin/GO/goSlimMapper.pl

2 https://www.yeastgenome.org/goTermFinder
