# Supplementary figures and images for: Genome-Wide Identification of Host Genes Required for Toxicity of Bacterial Cytolethal Distending Toxin in a Yeast Model
Source: Front Microbiol. 2019 Apr 26;10:890. doi: 10.3389/fmicb.2019.00890 (PMC6497811; doi:10.3389/fmicb.2019.00890)

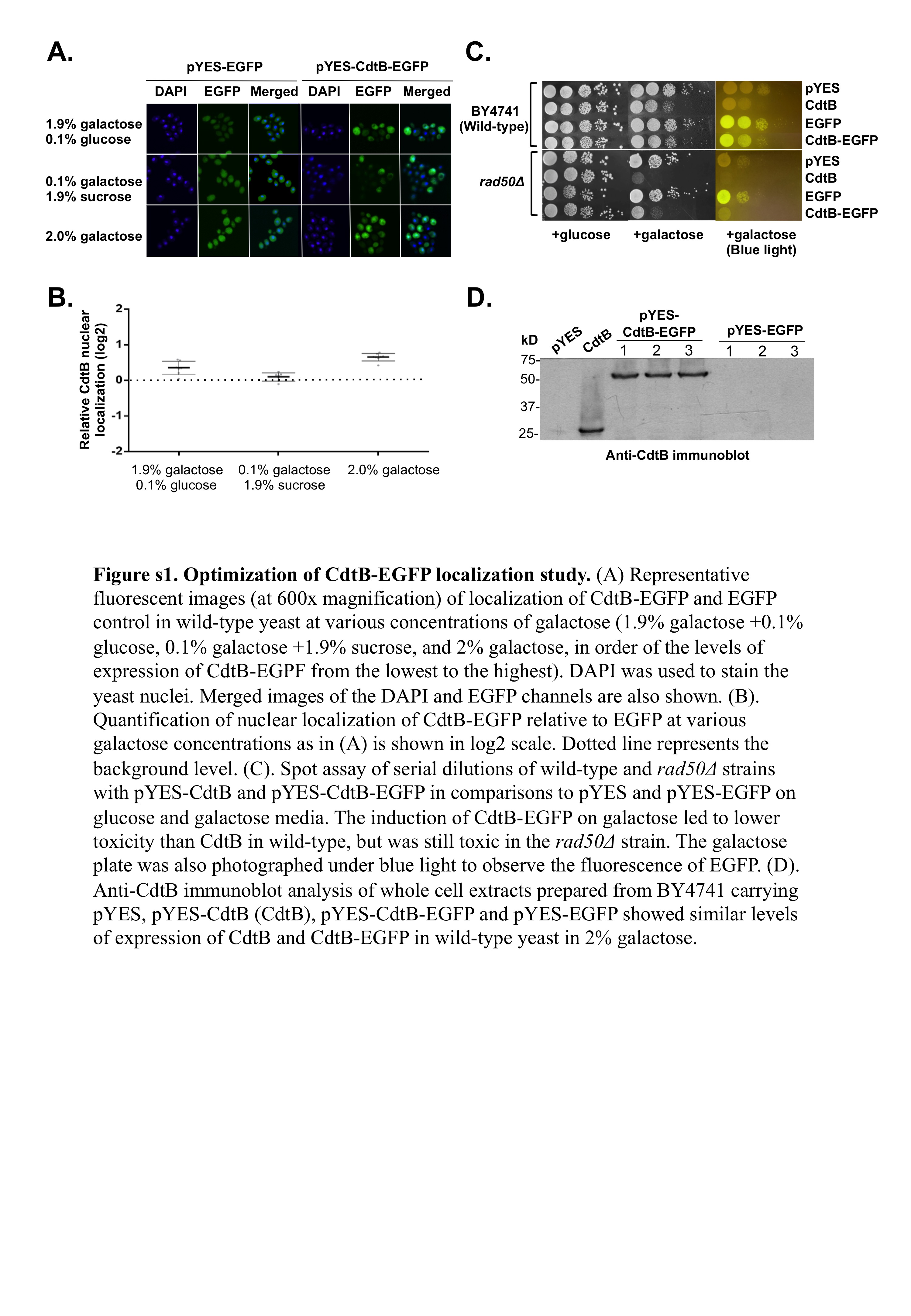

Supplement: Supplementary file 1 [file Image_1.jpg]

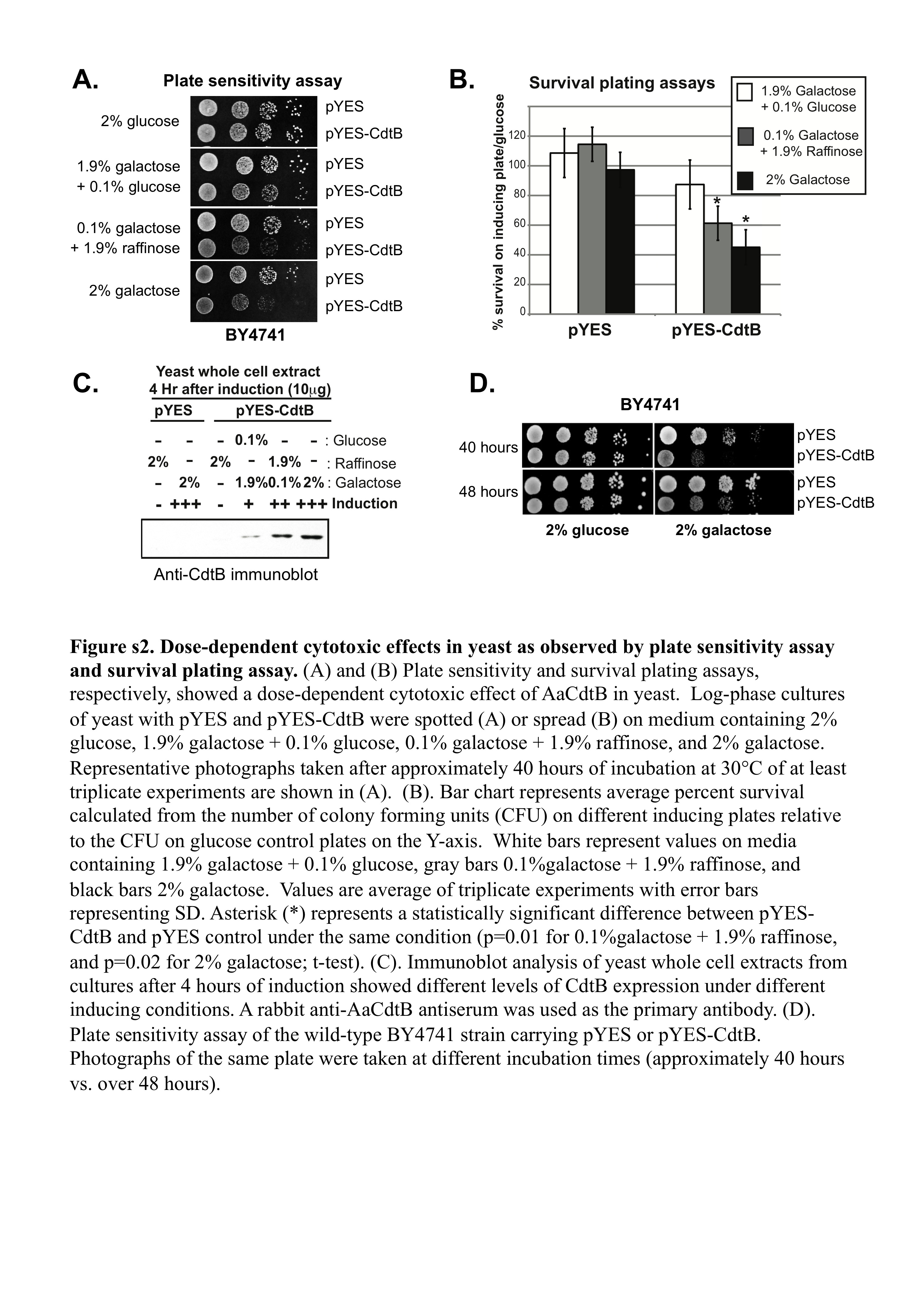

Supplement: Supplementary file 2 [file Image_2.jpg]
