# Supplementary material for: Genome-Wide Identification of Host Genes Required for Toxicity of Bacterial Cytolethal Distending Toxin in a Yeast Model
Source: Front Microbiol. 2019 Apr 26;10:890. doi: 10.3389/fmicb.2019.00890 (PMC6497811; doi:10.3389/fmicb.2019.00890)
Supplement: Supplementary file 3 [file Image_3.pdf]

Fig. s3 (A)

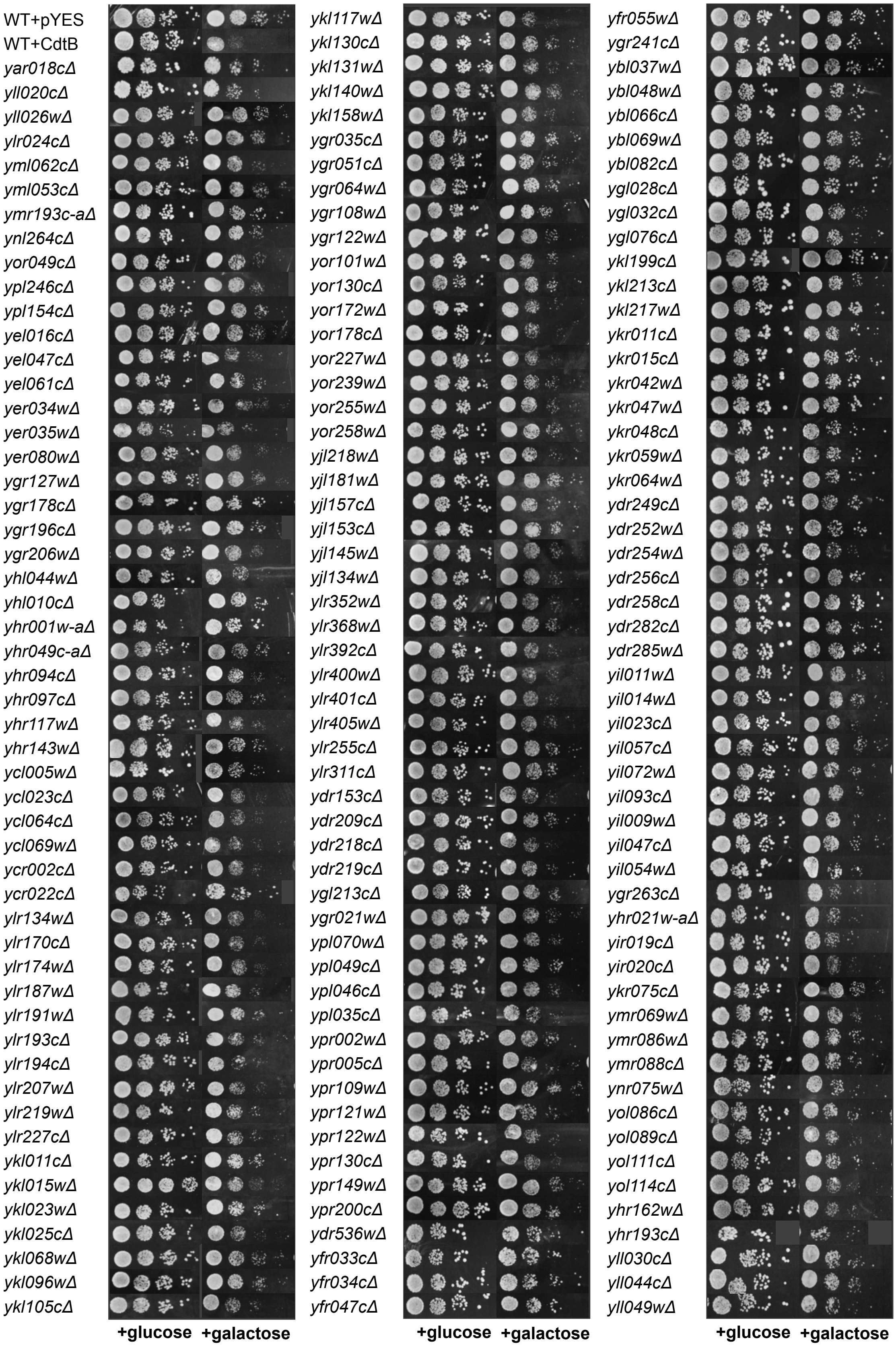
WT+pYES
WT+CdtB
yar018cΔ
yll020cΔ
yll026wΔ
ylr024cΔ
yml062cΔ
yml053cΔ
ymr193c-aΔ
ynl264cΔ
yor049cΔ
ypl246cΔ
ypl154cΔ
yel016cΔ
yel047cΔ
yel061cΔ
yer034wΔ
yer035wΔ
yer080wΔ
ygr127wΔ
ygr178cΔ
ygr196cΔ
ygr206wΔ
yhl044wΔ
yhl010cΔ
yhr001w-aΔ
yhr049c-aΔ
yhr094cΔ
yhr097cΔ
yhr117wΔ
yhr143wΔ
ycl005wΔ
ycl023cΔ
ycl064cΔ
ycl069wΔ
ycr002cΔ
ycr022cΔ
ylr134wΔ
ylr170cΔ
ylr174wΔ
ylr187wΔ
ylr191wΔ
ylr193cΔ
ylr194cΔ
ylr207wΔ
ylr219wΔ
ylr227cΔ
ykl011cΔ
ykl015wΔ
ykl023wΔ
ykl025cΔ
ykl068wΔ
ykl096wΔ
ykl105cΔ
+glucose +galactose
ykl117wΔ
ykl130cΔ
ykl131wΔ
ykl140wΔ
ykl158wΔ
ygr035cΔ
ygr051cΔ
ygr064wΔ
ygr108wΔ
ygr122wΔ
yor101wΔ
yor130cΔ
yor172wΔ
yor178cΔ
yor227wΔ
yor239wΔ
yor255wΔ
yor258wΔ
yjl218wΔ
yjl181wΔ
yjl157cΔ
yjl153cΔ
yjl145wΔ
yjl134wΔ
ylr352wΔ
ylr368wΔ
ylr392cΔ
ylr400wΔ
ylr401cΔ
ylr405wΔ
ylr255cΔ
ylr311cΔ
ydr153cΔ
ydr209cΔ
ydr218cΔ
ydr219cΔ
ygl213cΔ
ygr021wΔ
ypl070wΔ
ypl049cΔ
ypl046cΔ
ypl035cΔ
ypr002wΔ
ypr005cΔ
ypr109wΔ
ypr121wΔ
ypr122wΔ
ypr130cΔ
ypr149wΔ
ypr200cΔ
ydr536wΔ
yfr033cΔ
yfr034cΔ
yfr047cΔ
+glucose +galactose
yfr055wΔ
ygr241cΔ
ybl037wΔ
ybl048wΔ
ybl066cΔ
ybl069wΔ
ybl082cΔ
ygl028cΔ
ygl032cΔ
ygl076cΔ
ykl199cΔ
ykl213cΔ
ykl217wΔ
ykr011cΔ
ykr015cΔ
ykr042wΔ
ykr047wΔ
ykr048cΔ
ykr059wΔ
ykr064wΔ
ydr249cΔ
ydr252wΔ
ydr254wΔ
ydr256cΔ
ydr258cΔ
ydr282cΔ
ydr285wΔ
yil011wΔ
yil014wΔ
yil023cΔ
yil057cΔ
yil072wΔ
yil093cΔ
yil009wΔ
yil047cΔ
yil054wΔ
ygr263cΔ
yhr021w-aΔ
yir019cΔ
yir020cΔ
ykr075cΔ
ymr069wΔ
ymr086wΔ
ymr088cΔ
ynr075wΔ
yol086cΔ
yol089cΔ
yol111cΔ
yol114cΔ
yhr162wΔ
yhr193cΔ
yll030cΔ
yll044cΔ
yll049wΔ
+glucose +galactose

Fig. s3 (B)

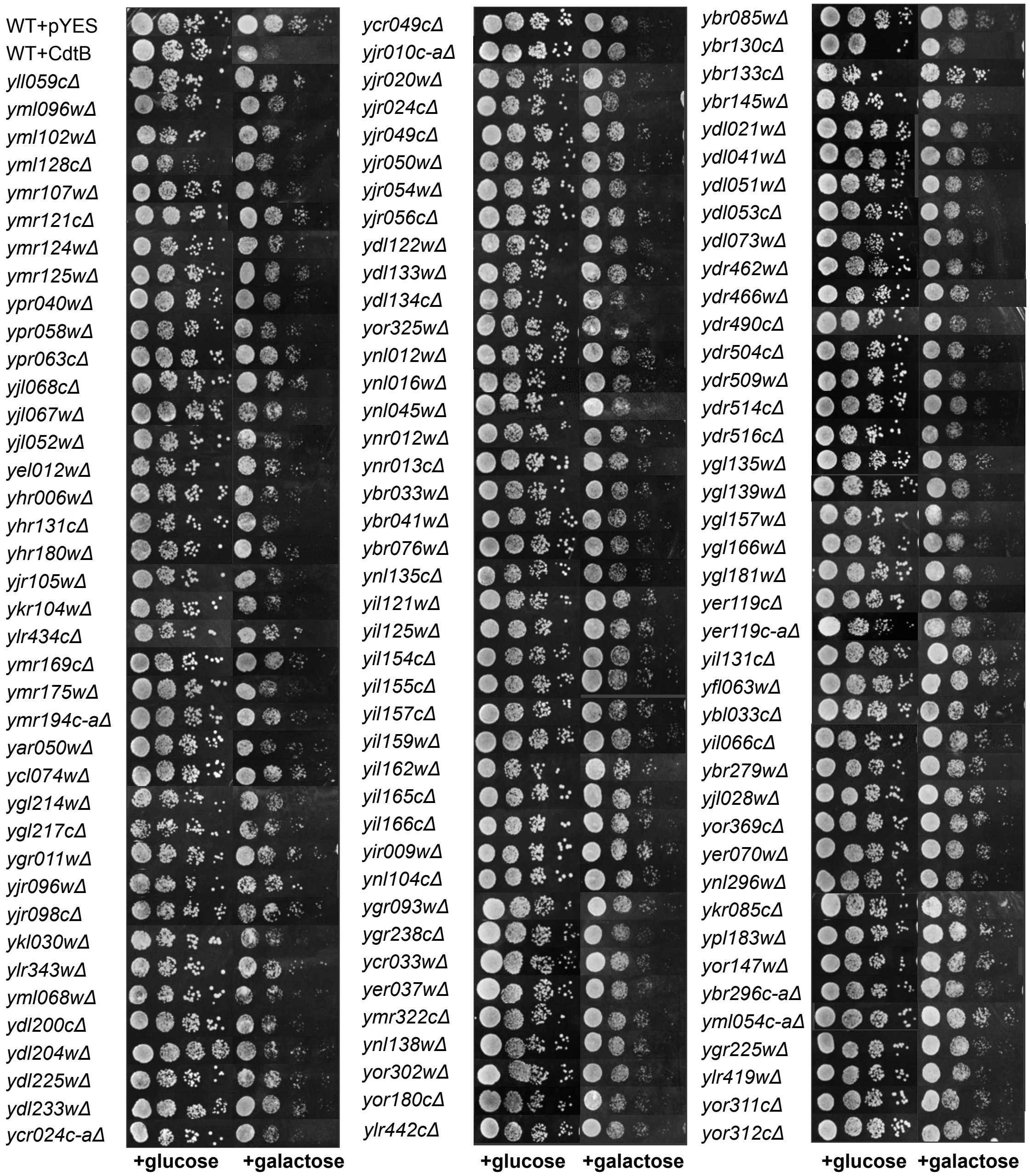

Figure s3. (A, B). CdtB susceptibility of 281 yeast deletion mutants that showed resistance to CdtB in comparison to the wild-type (WT; BY4741). All mutants, as listed by systematic ORF names, were transformed with pYES-CdtB and serial dilutions were spotted on solid media with galactose to induce CdtB expression and with glucose as controls. After approximately 40 hours of incubation at 30°C, these mutants showed more growth, at various levels in terms of number of colonies and colony sizes, than the wild-type when CdtB was induced.
