# Supplementary material for: Genome-Wide Identification of Host Genes Required for Toxicity of Bacterial Cytolethal Distending Toxin in a Yeast Model
Source: Front Microbiol. 2019 Apr 26;10:890. doi: 10.3389/fmicb.2019.00890 (PMC6497811; doi:10.3389/fmicb.2019.00890)
Supplement: Supplementary file 4 [file Table_1.docx]

| **Supplementary Table 1: Genes whose deletion confer CdtB resistance categorized by GO cellular components** | | | |
| --- | --- | --- | --- |
| **GO-Slim term** | **Cluster frequency** | **Genome frequency** | **Genes annotated to the term** |
| Cytoplasm | 159 out of 243 genes, 65.4% | 4309 of 6433 genes, 67% | *AAC3, ABP140, ADH1, ADH5, ADO1, AGA2, ALD3, ALG3, APL3, APS1, ARI1, ART10, AST1, AVT6, BNA6, BTT1,CAC2, CCE1, CDC10, CHA1, CLB1, COA1, COS10, CTA1, CWP1, DCI1, DRN1, DSE2, DUS3, EGD2, EMI2, ENT5,EPO1, FAT1, FKH1, FLC3, FLO11, FPR1, FRD1, GAC1, GPM2, GTS1, GUT2, HAL9, HER1, HNT3, HRD3, HSL7,HSP104, HSP78, IDP2, IMP2', INO1, JEN1, KGD1, LAP2, LCB3, LDB16, LDB18, LEU4, MDE1, MDM30, MDM32,MDY2, MFG1, MNT3, MPC2, MRPL20, MRPL28, MRX10, MSC1, MUK1, MVB12, NAP1, NAT4, NCE102, NCW2,NPP2, OAF3, ORT1, PBP1, PBP4, PDC5, PDH1, PDR17, PEP4, PEX13, PHO4, PHO91, PKH1, PPH21, PTH4, PUB1,QCR10, QCR6, RAS1, RBD2, RGI2, RIB1, RNR1, RNR3, RPL15B, RPL1B, RPS12, RSB1, RTC6, RTN2, SAY1, SBA1,SEG1, SFH5, SHE2, SHE3, SHS1, SIP18, SIR3, SKG3, SKI8, SPC1, SPG3, SPO1, SPR28, SRF1, SRV2, STL1, STP2, SYG1,TDH1, TIF1, TIR3, TOM71, UBC8, UBP1, UPS1, URK1, UTH1, UTR1, VBA1, VBA3, YAP1802, YDR514C, YGR021W,YGR122W, YHR097C, YHR131C, YIL166C, YJL028W, YJL068C, YJR056C, YJR098C, YKE4, YKL023W, YKR075C,YMC1, YML053C, YML096W, YPR063C, YPR109W, YRM1* |
| membrane | 68 out of 243 genes, 28% | 1629 of 6433 genes, 25.3% | *AAC3, ADH1, ALG3, APL3, APS1, AST1, AVT6, CCE1, CDC10, COA1, COS10, CWP1, ENT5, FAT1, FLC3, FLO11,FRD1, GUT2, HRD3, HXT1, JEN1, LCB3, LDB16, MDM32, MPC2, MRX10, MSC1, MVB12, NCE102, NCW2, NUP100,ORT1, OSW1, PDH1, PEX13, PHO91, QCR10, QCR6, QDR2, RAS1, RBD2, RRT1, RSB1, SAY1, SFH5, SHE2, SHS1,SPC1, SPO1, SPR28, SRF1, STL1, SYG1, TDH1, TGL1, TOM71, UPS1, UTH1, VBA1, VBA3, YAP1802, YGR122W,YHL044W, YIL166C, YKE4, YKL030W, YLR311C, YMC1* |
| nucleus | 65 out of 243 genes, 26.7% | 2208 of 6433 genes, 34.3% | *ADH5, ADO1, ARI1, BNA6, CAC2, CHL4, CLB1, COS10, CUP2, DIG1, DRN1, DUS3, ELC1, FKH1, FPR1, GTS1, HAL9,HNT3, HOP1, HSP104, ISY1, LAP2, LHP1, MDM30, MDY2, MFG1, MFT1, MGT1, MSL1, NAT4, NUP100, OAF3, PAF1,PBP1, PBP4, PDC5, PHO4, PPH21, PUB1, PUT3, QCR6, RAS1, RBD2, RIB1, RNR1, RPL15B, RTN2, SBA1, SFH5, SIR3,SKI8, SPO1, STO1, STP2, URK1, UTR1, YCL074W, YDR514C, YGR122W, YHR097C, YJR056C, YKR011C, YKR075C,YML053C, YRM1* |
| cellular component unknown | 51 out of 243 genes, 21% | 677 of 6433 genes, 10.5% | *AHK1, ARR2, DUS4, ECM12, ECM8, EDS1, ETP1, FAA3, ITT1, KIN3, PKH3, RBH1, RRT7, SEF1, SPG4, THI22,YCL023C, YCR022C, YCR049C, YDL041W, YDR209C, YDR249C, YDR509W, YER119C-A, YFL063W, YGL214W,YGL217C, YGR011W, YGR035C, YGR051C, YGR064W, YGR127W, YHR049C-A, YHR180W, YIL054W, YIR020C,YJL067W, YJL218W, YJR020W, YKL131W, YKR015C, YKR047W, YLL020C, YLL059C, YLR255C, YLR400W,YLR434C, YMR194C-A, YOR325W, YPL035C, YPR130C* |
| Mitochon-drion | 44 out of 243 genes, 18.1% | 1178 of 6433 genes, 18.3% | *AAC3, CCE1, CHA1, COA1, CTA1, FLC3, FPR1, FRD1, GUT2, HAL9, HER1, HSP78, JEN1, KGD1, LDB16, LEU4,MDM30, MDM32, MPC2, MRPL20, MRPL28, MRX10, MSC1, NCE102, OAF3, ORT1, PBP1, PDH1, PEP4, PTH4,QCR10, QCR6, RNR3, RTC6, SIR3, SYG1, TDH1, TOM71, UPS1, UTH1, YDR514C, YGR021W, YJR098C, YMC1* |
| Endo-membrane system | 34 out of 243 genes, 14% | 977 of 6433 genes, 15.2% | *AGA2, ALG3, APL3, APS1, AST1, COS10, ENT5, FAT1, FLC3, HRD3, LCB3, LDB16, MNT3, MSC1, MVB12, NCE102,NPP2, NUP100, PDR17, PEP4, RBD2, RSB1, RTN2, SAY1, SFH5, SHE2, SPC1, SPG3, SPO1, UBP1, VBA3, YKE4,YPR063C, YPR109W* |
| endoplasmic reticulum | 25 out of 243 genes, 10.3% | 615 of 6433 genes, 9.6% | *AGA2, ALG3, COS10, FAT1, FLC3, HRD3, LCB3, LDB16, MSC1, NCE102, NPP2, PEP4, RSB1, RTN2, SAY1, SFH5,SHE2, SPC1, SPG3, SPO1, UBP1, VBA3, YKE4, YPR063C, YPR109W* |
| vacuole | 22 out of 243 genes, 9.1% | 470 of 6433 genes, 7.3% | *AGA2, AVT6, CWP1, DSE2, FAT1, FLO11, JEN1, MNT3, NCW2, PEP4, PHO91, RSB1, SFH5, SPO1, SRF1, STL1, SYG1,TIR3, VBA1, VBA3, YIL166C, YMC1* |
| plasma membrane | 20 out of 243 genes, 8.2% | 395 of 6433 genes, 6.1% | *ADH1, APL3, AST1, FAT1, FLO11, FRD1, HXT1, JEN1, MSC1, NCE102, NCW2, QDR2, RAS1, RSB1, SFH5, STL1,SYG1, TDH1, YGR122W, YHL044W* |
| site of polarized growth | 16 out of 243 genes, 6.6% | 262 of 6433 genes, 4.1% | *ABP140, APL3, AXL1, BNR1, CDC10, EPO1, FLO11, GTS1, HSL7, NCW2, SHE2, SHE3, SHS1, SKG3, SRV2, YAP1802* |
| cellular bud | 15 out of 243 genes, 6.2% | 241 of 6433 genes, 3.7% | *APL3, AXL1, BNR1, CDC10, EPO1, FLO11, GTS1, HSL7, NCE102, NCW2, SHE2, SHE3, SHS1, SKG3, YAP1802* |
| Mitochon-drial envelope | 14 out of 243 genes, 5.8% | 355 of 6433 genes, 5.5% | *AAC3, CCE1, COA1, GUT2, MDM32, MPC2, MRX10, ORT1, PDH1, QCR10, QCR6, TOM71, UPS1, UTH1* |
| ribosome | 12 out of 243 genes, 4.9% | 357 of 6433 genes, 5.5% | *FRD1, HER1, MDY2, MRPL20, MRPL28, PTH4, RPL15B, RPL1B, RPS12, RTC6, SEG1, YJL028W* |
| cell cortex | 12 out of 243 genes, 4.9% | 147 of 6433 genes, 2.3% | *ABP140, CDC10, GTS1, HSL7, LDB18, PKH1, RTN2, SFH5, SHS1, SPR28, SRV2, YAP1802* |
| cytoskeleton | 10 out of 243 genes, 4.1% | 200 of 6433 genes, 3.1% | *ABP140, ADY4, CDC10, GTS1, HSL7, LDB18, SHS1, SPR28, SRV2, YAP1802* |
| cell wall | 9 out of 243 genes, 3.7% | 98 of 6433 genes, 1.5% | *AGA2, CWP1, DSE2, FLO1, NCW2, SCW11, SPR28, TDH1, TIR3* |
| cytoplasmic vesicle | 8 out of 243 genes, 3.3% | 237 of 6433 genes, 3.7% | *APL3, APS1, AST1, COS10, ENT5, MVB12, PDR17, RBD2* |
| chromosome | 7 out of 243 genes, 2.9% | 386 of 6433 genes, 6.0% | *CAC2, CHL4, HOP1, MFT1, PAF1, SIR3, SKI8* |
| peroxisome | 4 out of 243 genes, 1.6% | 76 of 6433 genes, 1.2% | *CTA1, DCI1, FAT1, PEX13* |
| Golgi apparatus | 4 out of 243 genes, 1.6% | 199 of 6433 genes, 3.1% | *APS1, AST1, MNT3, RBD2* |
| microtubule organizing center | 3 out of 243 genes, 1.2% | 73 of 6433 genes, 1.1% | *ADY4, HSL7, LDB18* |
| nucleolus | 3 out of 243 genes, 1.2% | 329 of 6433 genes, 5.1% | *LHP1, RPL15B, SIR3* |
| extracellular region | 2 out of 243 genes, 0.8% | 31 of 6433 genes, 0.5% | *DSE2, FLO11* |
| can not be mapped to a GO slim term | 2 out of 243 genes, 0.8% |  | *SEG2, YLR352W* |
| not yet annotated | 2 out of 243 genes, 0.8% |  | *YKL158W, YKL199C* |
